# Cerium Phosphate Nanoparticles: Synthesis, Characterization, Biocompatibility, Regenerative Potential, and Antioxidant Activity

**DOI:** 10.3390/molecules30193916

**Published:** 2025-09-28

**Authors:** Ekaterina V. Silina, Victor A. Stupin, Natalia E. Manturova, Elena L. Chuvilina, Akhmedali A. Gasanov, Olga I. Andreeva, Elena V. Korobko, Natalia V. Andreeva, Svetlana A. Dodonova, Daria D. Tkachenko, Dmitry Y. Izmailov, Natalia Y. Tabachkova, Yulia G. Suzdaltseva

**Affiliations:** 1 I.M. Sechenov First Moscow State Medical University (Sechenov University), 119991 Moscow, Russia; daryatkch@mail.ru (D.D.T.); info@chemilum.ru (D.Y.I.); 2 Pirogov Russian National Research Medical University, 117997 Moscow, Russia; stvictor@bk.ru (V.A.S.); manturovanatali@yandex.ru (N.E.M.); 3 LANHIT LLC, 105118 Moscow, Russia; chuvilina.elena@lanhit.ru (E.L.C.); akhmedali@lanhit.ru (A.A.G.); a.olga@lanhit.ru (O.I.A.); 4 Vavilov Institute of General Genetics, Russian Academy of Sciences, 119333 Moscow, Russia; alenavk@yahoo.com (E.V.K.); muha-05@bk.ru (N.V.A.); 5 Kursk State Medical University, Karl Marx St, 3, 305041 Kursk, Russia; dodonovasa@kursksmu.net; 6 National University of Science & Technology MISIS, 119049 Moscow, Russia; ntabachkova@gmail.com

**Keywords:** cerium phosphate, nanoparticles, rhabdophane, regeneration, wound healing, cell proliferation, antioxidants, mesenchymal stem cells, keratinocytes, fibroblasts

## Abstract

The aim of this study was to synthesize, characterize, and investigate the biomedical effects of nanoscale cerium phosphate obtained via different synthesis techniques, as well as to evaluate the influence of various CePO_4_ concentrations on cells involved in skin structure regeneration (human mesenchymal stem cells, keratinocytes, and fibroblasts) and establish their antioxidant properties. *Methods and Results:* Cerium(III) orthophosphate was obtained by precipitation with ammonium dihydrogen phosphate from a nitrate solution. By changing the initial concentrations of the solutions and the drying and annealing temperatures, the best conditions for obtaining nanosized phosphate powders were established. The structure of rhabdophane was obtained by X-ray diffraction analysis, and the particle sizes were measured by transmission electron microscopy. The particle sizes ranged from 2 to 10 nm in the transverse direction and 20 to 50 nm in the longitudinal direction. Studies on cell lines have shown a high level of safety, as well as the regenerative potential of CePO_4_ nanoparticles, which have a stimulating effect on the proliferation of MSCs at concentrations of 10^−2^ to 10^−3^ M for 48 h after application and stimulate the metabolism of human keratinocytes and fibroblasts at a wide range of concentrations (10^−3^ to 10^−5^ M). A dose-dependent antioxidant effect of small CePO_4_ nanoparticles at a concentration of 10^−2^ to 10^−5^ has been established, which is stronger than ascorbic acid. *Conclusions:* A method for obtaining cerium phosphate nanoparticles with beneficial biomedical effects was developed. The non-cytotoxicity and regenerative potential of CePO_4_ were established at a wide range of concentrations on different cell lines that are involved in the healing of skin wounds, as were their antioxidant properties.

## 1. Introduction

The global population continues to grow steadily and has already reached 8.24 billion people. Concurrently, the number of diseases is increasing worldwide. The UN’s 2024 forecast, published on July 11, revised the total global population growth of the Earth by 2100 to 10.2 billion (https://www.un.org/development/desa/pd/world-population-prospects-2024 and https://www.un.org/en/UN-projects-world-population-to-peak-within-this-century (accessed on 23 September 2025)). Myocardial infarctions with and without cardiogenic shock [1,2,3,4], cerebral strokes [5,6,7], cancer [8,9,10], and patients with type 2 diabetes mellitus with vascular and purulent complications [11,12] contribute to a significant increase in the number of chronically ill patients who require constant therapy. The increase in life expectancy with naturally increasing somatic and mental comorbidities also contributes significantly to this number [13,14]. The growing problems of treating an increasing number of patients with chronic diseases and disabilities are forcing the scientific community to constantly look for new, more effective, and less expensive treatment methods. Achieving this goal requires the development of new technologies and drugs that follow different principles. Therefore, the development of new drugs will always remain a highly relevant task.

Medications containing metal nanoparticles, especially rare-earth metals, have taken a prominent place in several areas of modern pharmacology [15,16,17,18,19,20,21]. This increasing interest is due to the discovery of beneficial biomedical effects, such as antioxidant, antibacterial, and regenerative properties. Within this area, nanoparticles of cerium salts are widely studied. Cerium dioxide (CeO_2_) is undoubtedly the leader in this scientific field. Unlike the nanoparticles of cerium oxide, cerium phosphate nanoparticles have been sparsely studied for applications in biology, medicine, and veterinary science. According to the Scopus international database’s search engine, there are 20,205 documents with the phrase [nano* AND cerium AND oxide*] in the title, abstract, or keywords, while there are only 803 publications with [nano* AND cerium AND phosphate*], which is 25 times less. According to the electronic biomedical library Pubmed, there are 3435 and 207 publications on cerium oxide and cerium phosphate, respectively. According to the library, the number of publications is 100 and 10 on cerium oxide and phosphate, respectively.

Cerium phosphate-based composites have been proven to be usable and are being used in various applications, such as measuring glucose and hydrogen peroxide levels [22,23], treating bone metastases from breast cancer [24], and as components in sunscreens [25,26,27]. However, the development of new cerium nanoparticle-based products has faced certain challenges.

The successful modeling of cerium phosphate/oxide nanoparticles as substances that simulate the action of enzymes and have beneficial biological effects on the body directly depends on the surface structure of the synthesized crystals, as noted in numerous studies [28,29,30,31,32]. In other studies, it has been shown that many factors that are insignificant or do not affect the synthesis process and outcomes in routine inorganic chemistry are crucial in the synthesis of cerium nanoparticles and nanocomposites, both in terms of the size and shape of the resulting nanoparticles [28,30,33,34,35] and their biological effects [35,36,37,38,39,40]. Therefore, we began our research by developing various methods for the synthesis of cerium orthophosphate and evaluating the physical characteristics and potential biological effects of the resulting nanoparticles in vitro.

The structural features, physical and chemical properties, and characteristics of rare-earth orthophosphates are actively being studied [41,42,43,44,45]. These compounds are promising for use in ion conductors, heat-resistant ceramics, and optical materials [46,47,48,49,50]. Their low toxicity and biocompatibility make it possible to use orthophosphates of rare-earth elements in the biomedical field [51,52,53].

Among the orthophosphates of rare-earth elements, cerium(III) orthophosphates—CePO_4_ (monazite structure) and CePO_4_·xH_2_O (rhabdophane structure)—occupy a special place. They are widely distributed as natural minerals, can be obtained synthetically [50,54,55,56,57], and are promising materials for application as UV filters [25,26,27]. The practical interest in cerium phosphate with a rhabdophane structure is due not only to the fact that it is a convenient synthetic precursor of monazite, but also to its higher reactivity (compared with monazite), which suggests that it can be successfully applied for biomedical purposes. Therefore, studies of the biological activity of CePO_4_ rhabdophane nanoparticles through in vitro model experiments are highly relevant and can have beneficial effects in regenerative medicine.

The purpose of this interdisciplinary work is to synthesize nanoscale cerium phosphate obtained under different synthesis conditions by precipitation with ammonium dihydrogen phosphate from aqueous solutions of cerium(III) nitrate to study the physicochemical characteristics and biomedical effects of cerium nanophosphate, with an assessment of the effect of various concentrations of CePO_4_ NPs sols (from 10^−2^ to 10^−5^ M) on the metabolic and proliferative activity of different cell cultures that are involved in wound healing (human mesenchymal stem cells, fibroblasts, and keratinocytes), as well as to establish their antioxidant properties for the subsequent creation of a regeneration-stimulating medical product.

## 2. Results

### 2.1. Results of Physicochemical Examinations

#### 2.1.1. Results of Transmission Electron Microscopy

**Figure 1:**
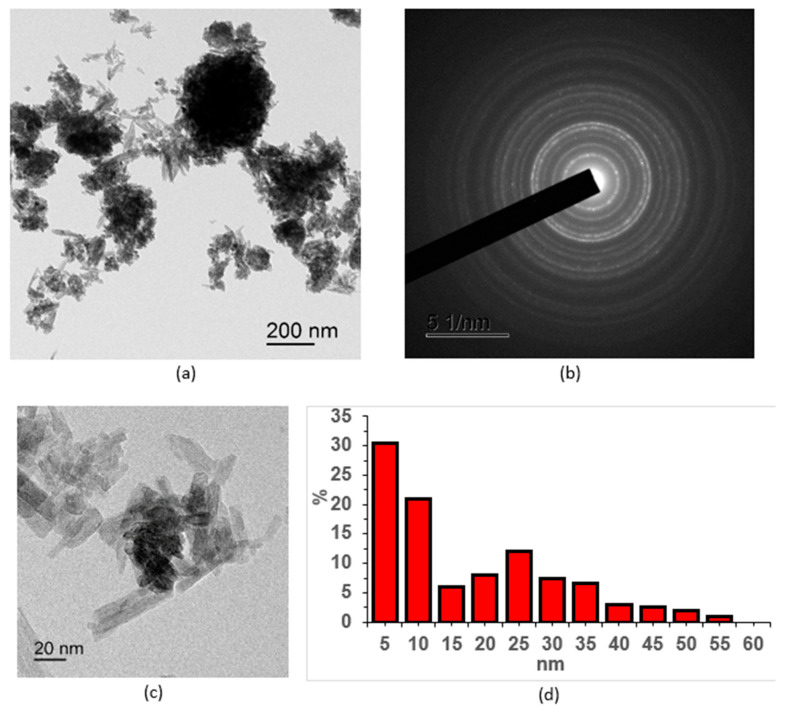
TEM images of the CePO_4_-I NPS sample: (**a**) overview image; (**b**) electron diffraction pattern; (**c**) magnified image of individual nanosticks; (**d**) particle size distribution.

**Figure 2:**
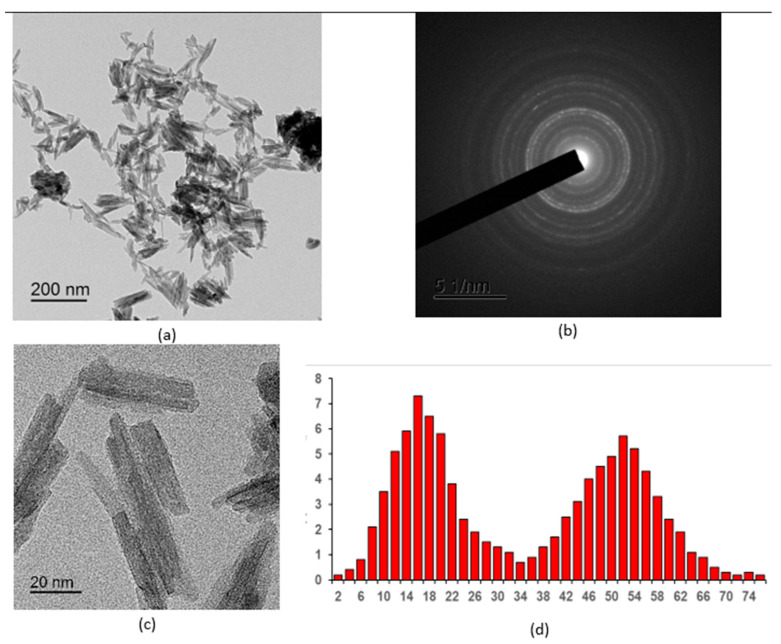
TEM images of the CePO_4_-II NPS sample: (**a**) overview image; (**b**) electron diffraction pattern; (**c**) magnified image of individual nanosticks; (**d**) particle size distribution.

**Figure 3:**
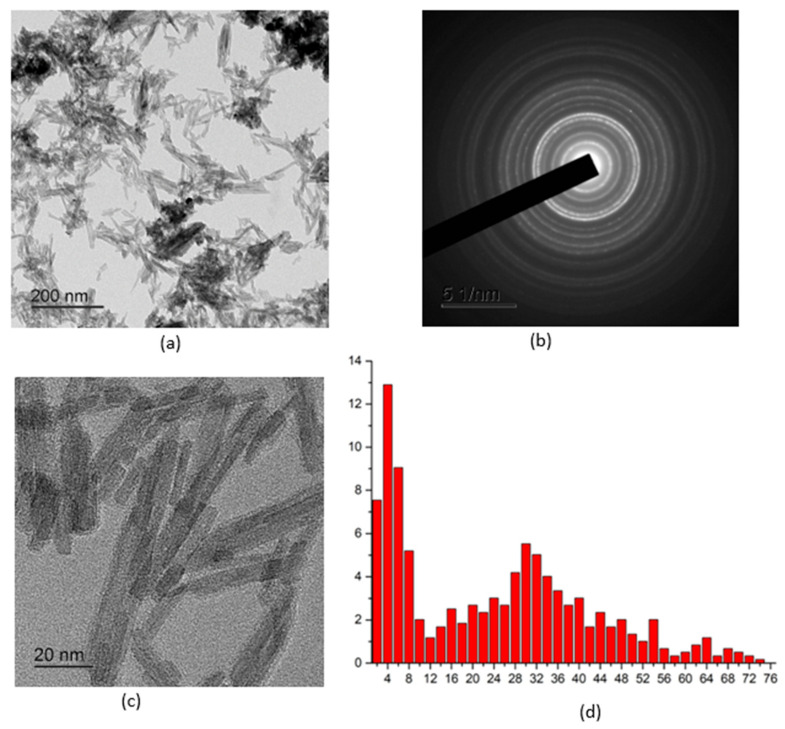
TEM images of the CePO_4_-III NPS sample: (**a**) overview image; (**b**) electron diffraction pattern; (**c**) magnified image of individual nanosticks; (**d**) particle size distribution.

**Figure 4:**
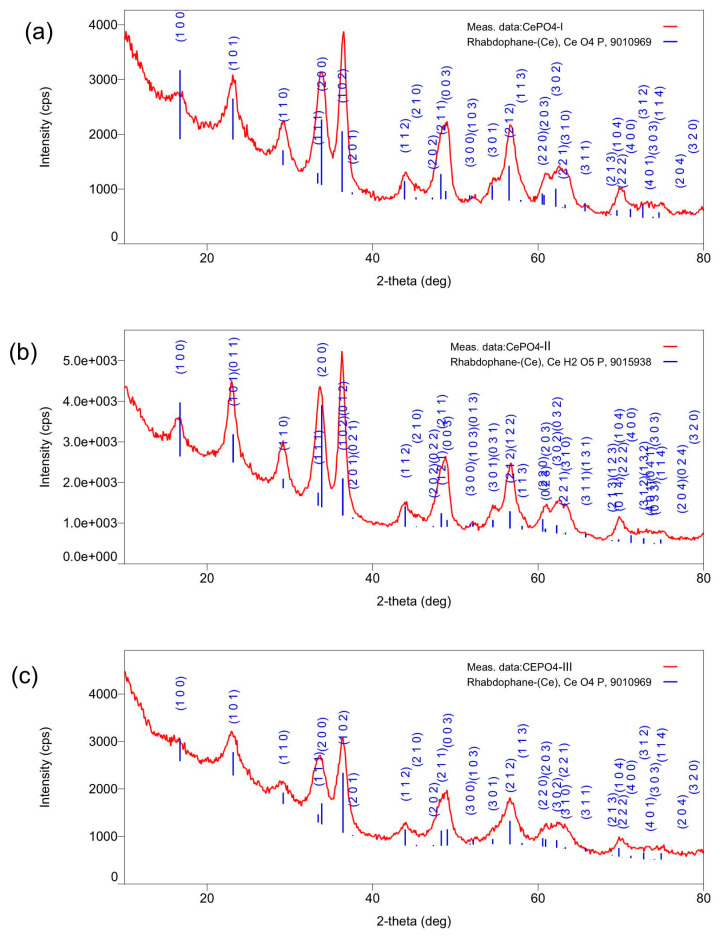
XRD of CePO_4_ samples: (**a**) CePO_4_-I, (**b**) CePO_4_-II, (**c**) CePO_4_-III. The single-phase nature of the samples and their correspondence to one structural type of rhabdophane was confirmed.

**Figure 5:**
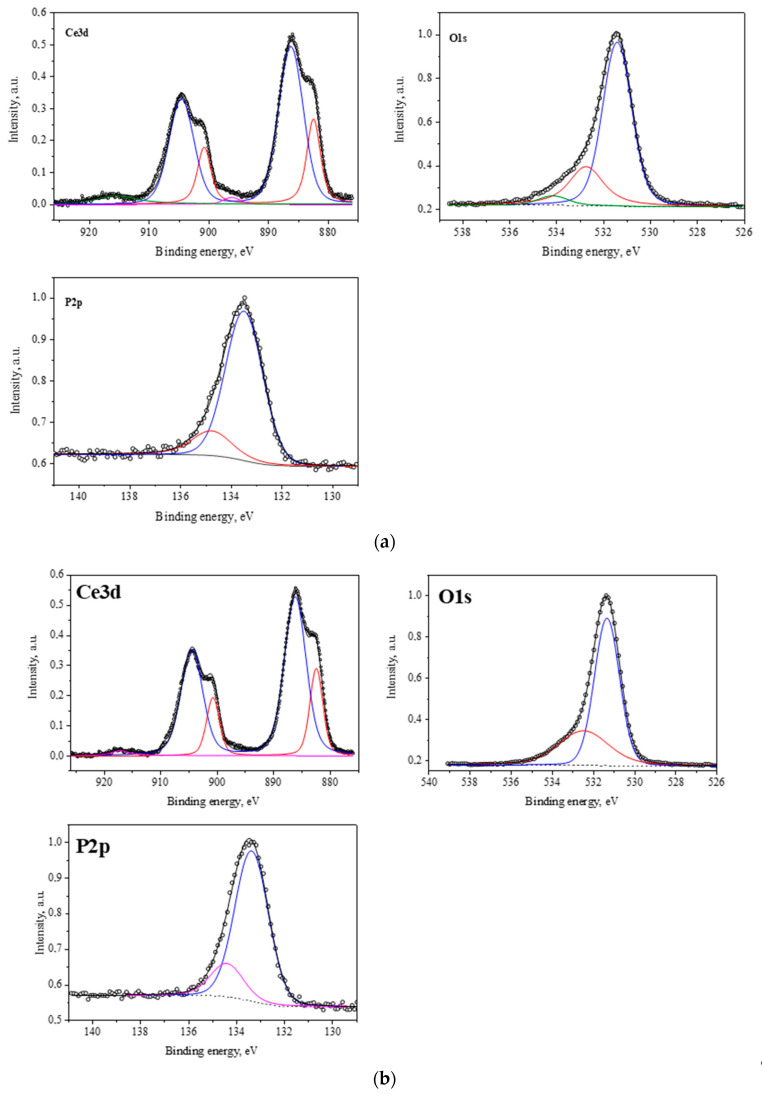

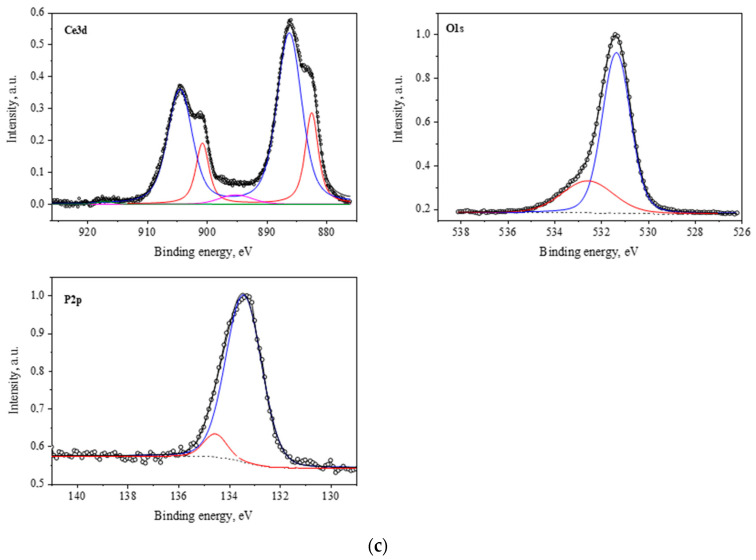
X-ray photoelectron spectra of the obtained cerium orthophosphate compounds in the Ce3d, O1s, and P2p regions: (**a**) CePO_4_-I, (**b**) CePO_4_-II, (**c**) CePO_4_-III.

Figure 1, Figure 2, Figure 3, Figure 4 and Figure 5 show high-resolution TEM images of polycrystalline grains, electron micrographs, and particle size distribution graphs for all the samples studied. According to the TEM data, all three samples of CePO_4_ powders have a similar appearance and consist of agglomerates of fine particles. The number of particles in the agglomerates varies widely. The size of the agglomerates ranges from hundreds of nanometers to a few microns. In addition to the agglomerates, individual nanoparticles are also present in the sample. The diffraction patterns in the selected region indicate that the powders are crystalline, which is consistent with the results of the X-ray diffraction analysis. The location of the diffraction maxima on the ring electronograms corresponds to the CePO_4_ phase of all three samples. The blurring of the rings on the electronogram also indicates the fine-grained structure of the powder. The particle shape is anisotropic and predominantly needle-like. When constructing the particle size distribution, the longitudinal and transverse dimensions of the particles were taken into account.

The TEM image of the CePO_4_-I sample (Figure 1) illustrates the crystalline morphology of the anisotropic particles, which is also confirmed by the diffraction pattern. The arrangement of the rings on the electron diffraction pattern corresponds to the CePO_4_ phase. The CePO_4_-I powder sample contained a large number of agglomerates that were up to micron nm in size. The individual crystals of the CePO_4_-I sample ranged in size from 5 to 60 nm. The average size of CePO_4_-I crystals was 10 nm (width) × 25 nm (length).

The CePO_4_-II sample (Figure 2) was less agglomerated than the CePO_4_-I sample, and its crystal size varied widely, from 5 to 80 nm. The crystals were also mostly rectangular, with an average size of 16 nm (width) × 52 nm (length).

Figure 3 shows a TEM image of CePO_4_-III powder particles. The powder consists of anisotropic rod-shaped particles of different lengths. The high-resolution images show that the anisotropic particles are crystalline. The images of atomic planes can be seen in the body of the particles. The crystallinity of the powder is also confirmed by the diffraction pattern. The arrangement of the rings on the electron diffraction pattern corresponds to the CePO_4_ phase. The size distribution is also bimodal. The thickness of the particles varies slightly, mainly between 2 and 6 nm. The maximum distribution of particle thickness by size corresponds to 4 nm (transverse size). The length of the particles varies between 12 and 75 nm, with a maximum distribution of 30 nm in the longitudinal size.

#### 2.1.2. Results of X-Ray Diffraction Analysis

According to the XRD data, all three samples of cerium phosphate powder are formed in the rhabdophane crystal structure and indicate the presence of only one phase—cerium(III) phosphate. The crystallite sizes indicate the polycrystalline nature of the samples. The interplanar spacings are in good agreement with the plane families from standard CePO_4_ cards. The microstrain values also confirm the polycrystalline nature of the samples. Some blurring of peaks in the X-ray spectrum of the CePO_4_-III sample seem to be related to insufficient crystal formation, which may be due to a reduction in the annealing time and temperature of the samples (Table 1; Figure 4).

#### 2.1.3. Results of X-Ray Photoelectron Spectroscopy

To determine the chemical composition, the degree of oxidation of the metal cation and the ratio of atomic concentrations of elements on the surface of the samples, the regions Ce(3d3/2), Ce(3d5/2), P(2p3/2), and O(1s) were measured. All three spectra were similar, and their shapes for Ce(3d) corresponded to the structure of Ce(3d3/2) and Ce(3d5/2) lines of cerium for cerium phosphate. The number of Ce(3d) peaks, their shape, and energy corresponded to the electronic state of Ce^3+^. In addition, the bond energies for Ce(3d), P(2p3/2), and O(1s) were in good agreement with the corresponding values from the databases for CePO_4_ (Table 2; Figure 5).

Thus, the physical and chemical methods of characterization of the synthesized nanopowders showed that the nanosized cerium phosphate obtained under industrial conditions has a clearly defined crystalline structure of cerium orthophosphate (CePO_4_), as well as a rhabdophane structure that is largely agglomerated and has a wide range of linear dimensions of individual grains.

### 2.2. Results of Culture Studies on Human Mesenchymal Stem Cells

#### 2.2.1. Monitoring MSC Growth Curves Using Real-Time Cell Analysis (RTCA)

**Figure 6:**
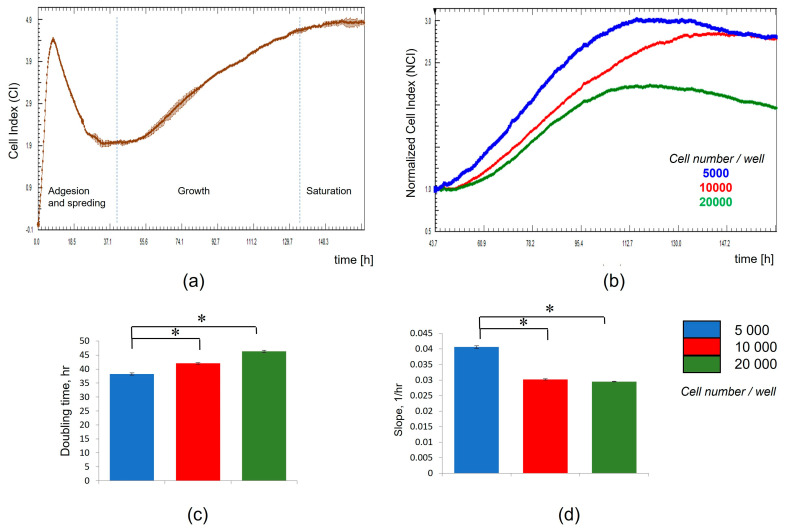
Optimization of MSC culture conditions using cell impedance measurements according to cell density at seeding. (**a**) MSC growth curves showing the phases of the cell growth: cell adhesion and spreading, cell growth phase, and stationary phase. (**b**) Normalized CI values for MSCs seeded at different cell densities (from 5000 to 20,000 cells per well). Curves represent the mean Cell Index value from 4 wells ± SD. (**c**) Doubling time parameter for MSCs seeded at different cell densities in the interval from 72 to 98 h. (**d**) Slope parameter of MSCs seeded at different cell densities in the interval from 72 to 98 h. Data are represented as mean of n = 4 measurements ± SD. *t*-test, *—reliability of differences at *p* < 0.05.

Growth curves of MSCs, based on cellular impedance measurements using the x-CELLigence system, were established according to the number of cells that were seeded in E-plates (from 5000 to 20,000 cells per well) to obtain the optimal cellular density. CI values were recorded in real time for a period of up to 160 h. As a whole, the impedance-based profiles showed the three phases of the cell growth curve (cell adhesion and spreading, cell growth phase, and stationary phase) (Figure 6a). After adhesion and spreading of cells, the CI values increased during the cell growth stage to reach the stationary phase. The CI values increased faster for MSCs that were seeded at a cell density of 5000 cells per well than for wells with higher initial cell densities (Figure 6b). The bar chart presented in Figure 6c shows the time required for a CI value to double. The doubling time parameter in the interval from 72 to 98 h was lower for MSCs that were seeded at a cell density of 5000 cells/well than for wells with a higher cell density. In contrast, the slope parameter corresponding to the growth curves of MSCs that were seeded at a cell density of 5000 cells/well was higher when a higher number of cells were seeded under the same conditions (Figure 6d). Thus, a cellular density of 5000 cells/well was selected as the optimum for cell seeding.

#### 2.2.2. Monitoring of CePO_4_-Induced Responses in MSCs Using RTCA

**Figure 7:**
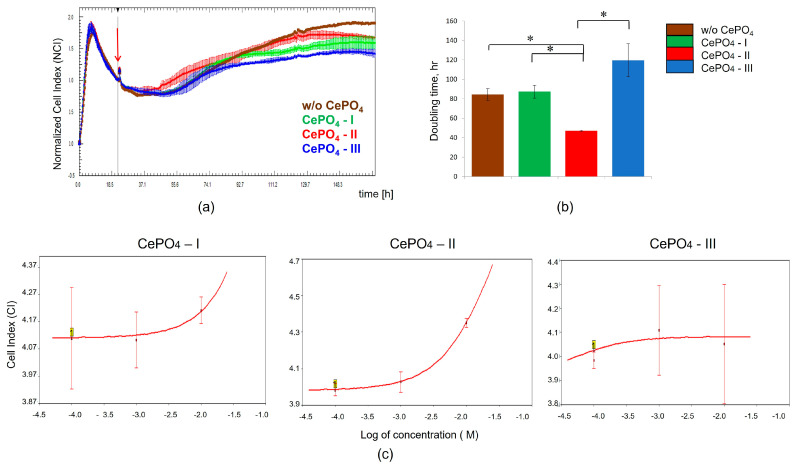
Real-time impedance analysis of MSCs treated with CePO_4_ NPs. (**a**) Response profiles of MSCs treated with 10^−2^ M CePO_4_-I, CePO_4_-II, and CePO_4_-III. The red arrow indicates the time after which the CePO_4_ samples were added to the MSCs. Curves represent the mean CI value from 4 wells ± SD. (**b**) CI doubling time for MSCs treated with 10^−2^ M CePO_4_ (I, II, III) in the interval from 24 to 48 h after CePO_4_ NP application. (**c**) The dose–response curves for CePO_4_ (I, II, III) at 48 h after MSC treatment. Data are represented as mean of n = 4 measurements ± SD. *t*-test, *—reliability of differences at *p* < 0.05.

To evaluate the cells’ response to different CePO_4_ samples (I, II, III), the cellular impedance of MSCs that were treated with or without titrated concentrations of CePO_4_ was measured in real time using the xCELLigence system. For this purpose, MSCs were seeded on E-plates at a cell density of 5000 cells per well. After overnight culture, the cells were attached and spread along the bottom. Then 10^−2^ M, 10^−3^ M, 10^−4^ M, and 10^−5^ M of CePO_4_-I, CePO_4_-II, and CePO_4_-III were added to cells in four duplications, and CI values were measured every 30 min for 170 h. Intact MSCs were used as controls. Figure 7a shows the response profiles of MSCs that were treated with 10^−2^ M of CePO_4_-I, CePO_4_-II, and CePO_4_-III. Longitudinal data of the normalized CI showed that CePO_4_-II application resulted in increased MSC proliferation compared with the control over 48 h after treatment, while CePO_4_-I and CePO_4_-III had no effect on cells during the same time period. However, the subsequent cumulative effect of CePO_4_-I, CePO_4_-II, and CePO_4_-III at a concentration of 10^−2^ M on MSCs had a negative impact on their proliferative activity. At the same time, it should be noted that none of the CePO_4_ samples (I, II, III) had a cytotoxic effect on MSCs and did not affect cell viability. The CI doubling time in the interval from 24 to 48 h after CePO_4_ application was lower for MSCs that were treated with 10^−2^ M CePO_4_-II, while the CI doubling time in samples that were treated with CePO_4_-I and CePO_4_-III at the same concentration had similar values to the intact cells (Figure 7b). The response profiles of MSCs that were treated with CePO_4_-I, CePO_4_-II, and CePO_4_-III at concentrations of 10^−3^ M, 10^−4^ M, and 10^−5^ M were similar to those of intact cells. The dose–response curves for CePO_4_ preparations at 48 h are shown in Figure 7c. Thus, our results demonstrated that at the applied concentrations, CePO_4_-I, CePO_4_-II, and CePO_4_-III do not affect cell viability or exert cytotoxic effects on MSCs. CePO_4_-II at a concentration of 10^−2^ M has a stimulating effect on the proliferative activity of MSCs during the first 48 h after application, presenting an advantage over its counterparts, CePO_4_-I and CePO_4_-III. With a longer exposure, CePO_4_-I, CePO_4_-II, and CePO_4_-III at a concentration of 10^−2^ M may have a negative effect on the proliferation of MSCs (most concerns relate to the CePO_4_-III sample). Taking the latter into account, a concentration of 10^−3^ M is preferable for biomedical research on cell lines, because it does not exhibit negative cumulative effects.

### 2.3. Results of Culture Studies on Human Fibroblasts

**Figure 8:**
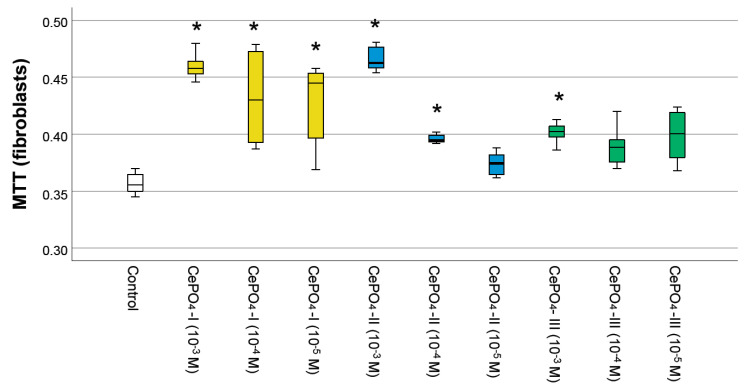
Effects of different synthesis options and concentrations of cerium phosphate nanoparticles on the metabolic activity of human fibroblasts in the MTT test (*—difference from control at *p* < 0.05; Kruskal–Wallis test). White is the control, yellow—CePO_4_-I, blue—CePO_4_-II, and green CePO_4_-III.

The results of the MTT test (Figure 8) showed that none of the variants of CePO_4_ had toxic effects on human fibroblast cultures at concentrations of 10^−3^–10^−5^ M. In all cases, the metabolism of fibroblasts was stimulated by cerium orthophosphate nanoparticles (by an average of 5–30%), and the highest OD value was observed at a concentration of 10^−3^ M in all samples, which was significantly different from the control. The maximum OD value was recorded for the CePO_4_-II sample at a concentration of 10^−3^ M (the OD value was 1.30 times higher than in the control, *p* < 0.05), while the same CePO_4_-II sample at a concentration of 10^−4^ M stimulated fibroblast metabolism by an average of 1.11 times (*p* < 0.05). All concentrations of the CePO_4_-I sample (10^−3^–10^−5^ M) stimulated the metabolism of fibroblasts by an average of 1.21–1.29 times compared with the control (*p* < 0.05). The OD value in co-culture of human fibroblasts with the CePO_4_-III sample was higher than the control by an average of 1.13 times (*p* < 0.05) at concentrations of 10^−3^–10^−5^ M.

**Figure 9:**
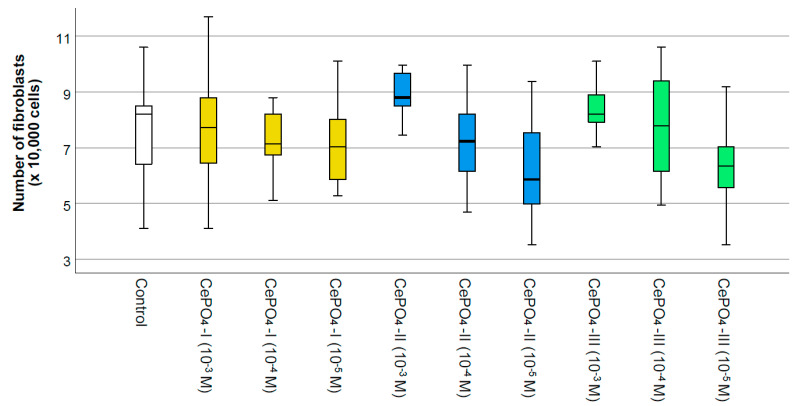
The effect of the methodology of the production synthesis of nanoceria and its concentration on the proliferative activity of fibroblasts when the cells are counted directly using an automatic cell counter.

**Figure 10:**
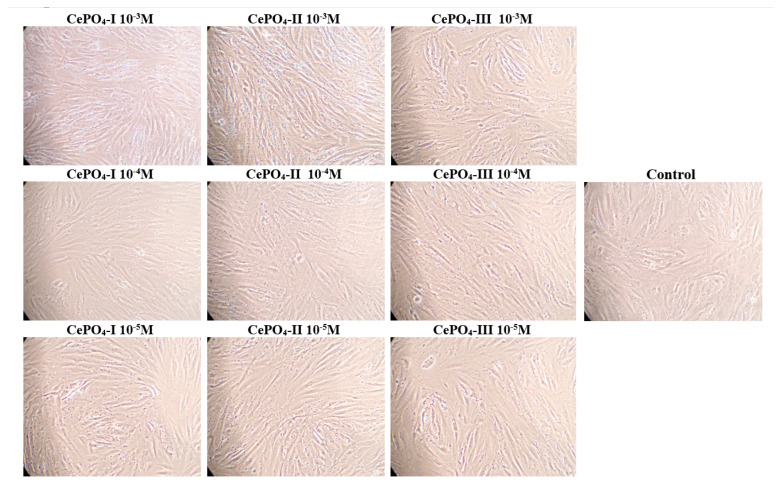
Photographs of human fibroblasts after 72 h of co-cultivation with CePO_4_ following different synthesis modifications and at different concentrations compared with control (magnification × 20).

A count of the number of fibroblasts after 72 h of co-cultivation did not reveal any significant differences from the control, which on the one hand indicates the absence of cytotoxicity, while on the other hand indicating the absence of a significant stimulating effect on fibroblast proliferation. A tendency towards increased cell proliferation was only observed in the CePO_4_-II sample at a concentration of 10^−3^ M and in the CePO_4_-III sample at a concentration of 10^−3^ M (if the median is 1.07 times higher than the control, *p* > 0.05; if the average is 1.17 times higher, then CePO_4_-III at 10^−3^ M is 1.19 times higher, *p* > 0.05) (Figure 9 and Figure 10).

It is important to note that no dead cells were detected at any concentration of any CePO_4_ sample, indicating that there was no cytotoxic effect.

Thus, the results of studies on human fibroblasts and keratinocytes showed the biocompatibility of all studied cerium phosphate samples at all concentrations. Although our studies on human fibroblasts did not reveal a clear advantage of any of the samples, they showed that the greatest stimulation of fibroblasts occurs at a concentration of 10^−3^ M in all samples, while a tendency for the greatest stimulation, metabolism, and proliferation was recorded in the sample of CePO_4_-II at a concentration of 10^−3^ M.

### 2.4. Results of Culture Studies on Human Keratinocytes

**Figure 11:**
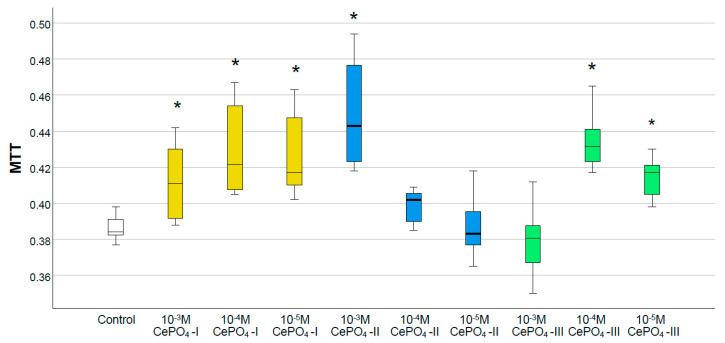
Effects of different synthesis options and concentrations of cerium phosphate nanoparticles on the metabolic activity of human keratinocytes in the MTT test (*—difference from control at *p* < 0.001; Kruskal–Wallis test).

The results of the MTT test showed a statistically significant increase in the OD value when human keratinocytes were co-cultured with CePO_4_-I at concentrations of 10^−4^ to 10^−5^ M (by 10–11%), with CePO_4_-II at a concentration of 10^−3^ M (by an average of 1.16 times), and with CePO_4_-III at a concentration of 10^−4^ M (by 1.12 times that of the control group, *p* < 0.01). The cerium phosphate samples did not exhibit any inhibitory effect on keratinocyte metabolism (significant decrease in OD) at any of the studied concentrations, which confirmed the non-toxicity and compatibility of all the studied nanoparticles (Figure 11).

**Figure 12:**
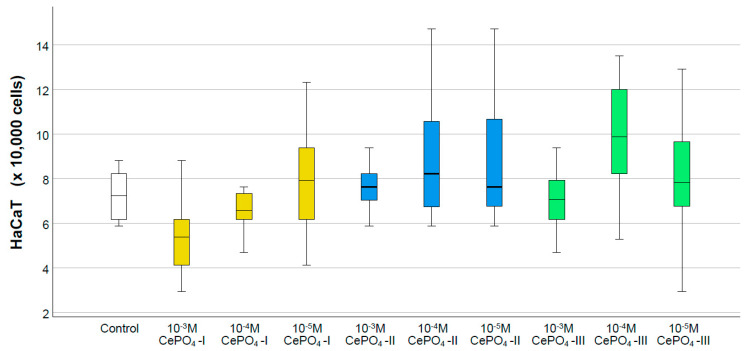
Results of counting the number of human keratinocytes during co-cultivation with cerium orthophosphate nanoparticles at different concentrations.

The counting of keratinocytes also showed that none of the ceria orthophosphates we studied had negative effects on the proliferative activity of HaCaT cells. Despite the fact that no statistically significant differences were found between the control and study groups, all concentrations of the CePO_4_-II sample were above the normal range (1.05–1.25 times on average), while CePO_4_-I was at 10^−5^ (1.09 times) and CePO_4_-III was at 10^−4^ M (1.36 times). The maximum cell number was recorded in the CePO_4_-III sample at concentrations of 10^−4^ M (an average of 36% or 1.36 times higher than the control) and at concentrations of 10^−4^–10^−5^ M in the CePO_4_-II sample (an average of 25% and 24% higher than the control, respectively) (Figure 12).

**Figure 13:**
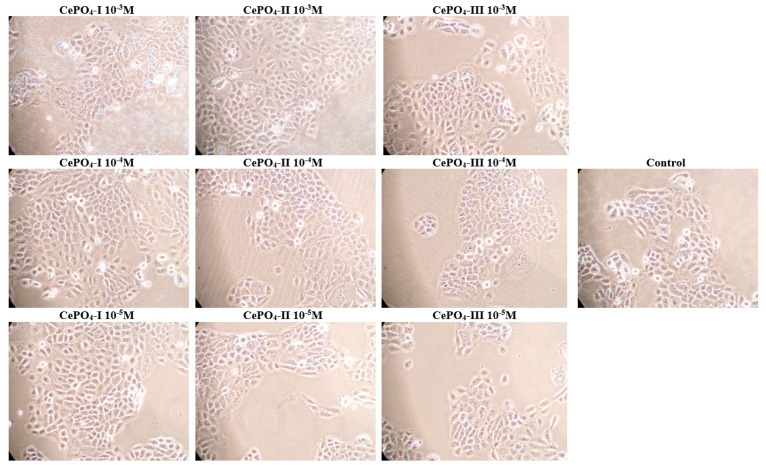
Photographs of human keratinocytes after 72 h of co-cultivation with CePO_4_ with different synthesis modifications and at different concentrations, compared with control (magnification × 20).

There were no dead keratinocytes in any of the CePO_4_ samples at any concentration. This demonstrates the non-cytotoxicity of cerium orthophosphates (Figure 13).

Based on the results of our studies, it can be concluded that to achieve the greatest stimulation of the proliferative and metabolic activity of keratinocytes, it is preferable to use CePO_4_-II at a concentration of 10^−3^ M, which has the greatest effect on the metabolism of keratinocytes, and CePO_4_-III at a concentration of 10^−4^ M, which has the greatest effect on the proliferation of keratinocytes and their metabolism.

### 2.5. Antioxidant Activity Results

The intensity of chemiluminescence in the Fenton reaction was studied using the best CePO_4_-II sample based on the results of our culture studies.

**Figure 14:**
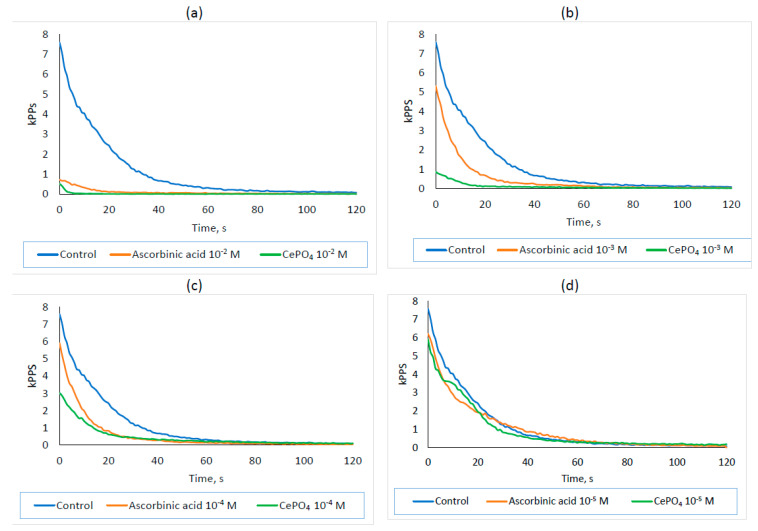
Kinetic profile of chemiluminescence of the reference experiment (control, blue line) and experiments with the addition of equal concentrations of cerium phosphate nanoparticles (green line) and the comparison sample with antioxidant ascorbic acid (orange line) at concentrations of (**a**) 10^−2^ M; (**b**) 10^−3^ M, (**c**) 10^−4^ M, and (**d**) 10^−5^ M.

During the study, it was found that cerium phosphate nanoparticles showed greater antioxidant activity than ascorbic acid at concentrations of 10^−2^, 10^−3^, 10^−4^, and 10^−5^ M (Table 3; Figure 14). The effect was dose-dependent and most pronounced at a concentration of 10^−2^ M. The integral light sum of the chemiluminescence reaction was on average 11.0 times higher in the control than in ascorbic acid at a concentration of 10^−2^ M (*p* < 0.05) and 44.6 times higher than CePO_4_ at a concentration of 10^−2^ M (*p* < 0.05). The difference between the ascorbic acid and CePO_4_ groups was on average 4.1-fold and statistically significant (*p* < 0.05), demonstrating greater antioxidant power in CePO_4_ NPs.

At a 10^−3^M concentration, ascorbic acid exhibited weaker antioxidant properties. The light sum in the control was 2.3 times higher on average than in ascorbic acid at 10^−3^ M (*p* > 0.05) and 10.2 times higher than CePO_4_ at 10^−3^ M (*p* < 0.05). The difference between the ascorbic acid and CePO_4_ groups was statistically significant (*p* < 0.05), with an average of 4.4 times the antioxidant capacity of CePO_4_ nanoparticles at a concentration of 10^−3^ M. At 10^−4^ M, the ascorbic acid and CePO_4_ NPs also showed antioxidant properties, with differences from the control averaging 2.0 times (*p* < 0.05) and 2.5 times (*p* < 0.05), respectively. Even at a concentration of 10^−5^ M, the CePO_4_-II NPs demonstrated antioxidant activity, with an average difference of 1.1 times (*p* < 0.05) compared with the control, while no antioxidant properties were observed for ascorbic acid at the same concentration.

Thus, CePO_4_-II NPs at concentrations of 10^−2^, 10^−3^, 10^−4^, and 10^−5^ have pronounced antioxidant activity that is stronger than that of ascorbic acid at an equivalent concentration. Nanoparticles have a dose-dependent antioxidant effect (like ascorbic acid), with the greatest effect occurring at 10^−2^ M (44.6 times), and a weak effect at 10^−5^ M (1.1 times).

## 3. Discussion

As a result of our experiments, the best of three modifications of the synthesis of cerium (III) orthophosphate—obtained by precipitation of ammonium dihydrogen phosphate from a solution of cerium (III) nitrate under similar conditions—was searched for to develop a new type of drugs based on them. Differences in the initial concentrations of solutions, the presence of a crystallization regulator, and the temperature of the washing liquid did not significantly affect the morphology of the resulting particles. However, the presence of a NH_4_NO_3_ crystallization regulator led to a slight increase in the size of the resulting cerium (III) phosphate particles, in contrast to the effect exerted by it during the isolation of cerium oxide [35]. In our opinion, the best reactivity of the material is achieved and its nanodispersed dimension is preserved the most at a temperature of 200 °C. The heat treatment of cerium phosphate, which is necessary to remove the bulk of the bound water, was carried out at 200 °C. Higher annealing temperatures contribute to the formation of larger and more perfect crystals, which naturally reduces their chemical (and possibly biological) activity. At temperatures below 200 °C, the complete dehydration of lanthanide phosphates means that LnPO_4_^.^·0.67H_2_O does not occur [58].

The physicochemical methods of characterization of the synthesized nanopowders (TEM, XRD, and XPS) in three groups showed that the nanosized CePO_4_ obtained under industrial conditions has a distinct crystalline structure of cerium(III) orthophosphate (CePO_4_), a rhabdophane structure, and an anisotropic shape ranging from spherical to rectangular nanosticks. The powders mainly contained nanosticks, with a wide range of linear dimensions (at least 2 × 10 nm). Since most researchers believe that the maximum effects in any biological object can only be achieved with nanoparticle sizes of no more than 20 nm [59,60,61], by maximizing the number of active quantum dots, the presence of such small phosphates was achieved, which is very encouraging. The practical interest in cerium phosphate with a rhabdophane structure (Rhabdophane) is due not only to the fact that it is a convenient synthetic precursor for monazite, but also to its higher (compared with monazite) reactivity, which suggests its potential use for biomedical applications, particularly as an inorganic UV filter in photoprotective formulations. In a recent study, it was demonstrated that the obtained rhabdophane nanoparticles were non-cytotoxic to human keratinocyte cells. Based on the above, prepared rhabdophane can be evaluated as a promising UV filter in photoprotective formulations [26].

Studies of aqueous suspensions of CePO_4_ NPs of a minimal size in all three groups and on different cell lines showed non-cytotoxicity and high regenerative potential. CePO_4_ nanoparticles had a stimulating effect on the proliferation of MSCs at concentrations of 10^−2^–10^−3^ M within 48 h after application. Stimulation of the metabolism of keratinocytes and human fibroblasts was recorded at all applied concentrations (10^−3^–10^−5^ M), including the minimum studied concentration. It is possible that differentiated cells are more responsive to the presence of nanoparticles in their environment.

The xCELLigence biosensor technology has numerous applications in fundamental research and clinical drug development, providing the possibility of real-time detection of cell responses to various stimuli in vitro [62,63].

RTCA has a clear advantage over endpoint methods and allows for label-free and non-invasive determination of the functional status of adhesive cells at different time points in the same well. This is particularly relevant when dealing with human primary cells, where the onset and duration of responses may vary significantly. MSCs can be isolated from various tissue sources and successfully expanded in vitro. All proposed MSC populations only grow in vitro as adherent cells. MSCs are considered a promising therapeutic agent because they possess multiple paracrine and immunomodulatory activities, providing protection against chronic inflammation and promoting tissue repair [64,65,66,67,68].

To evaluate the MSCs’ response to the application of different CePO_4_ preparations, we performed RTCA using the xCELLigence system. Our results revealed that CePO_4_ NPs at the applied concentrations do not cause damage or death of MSCs, which would be manifested by a progressive, sustained decrease in the CI [63,69]. Thus, CePO_4_ preparations do not exert a cytotoxic effect on MSCs.

The application of 10^−2^ M CePO_4_-II to MSCs resulted in increased cell proliferation compared with the control within 24–48 h after treatment. However, this effect was subsequently leveled. Our results indicate that the stimulating effect of CePO_4_-II on MSC proliferation is transient. CePO_4_-II showed a better effect on MSC proliferation compared with its counterparts, CePO_4_-I and CePO_4_-III. On the other hand, we have shown that long-term exposure to CePO_4_ (I, II, III) preparations can suppress the functional activity of MSCs. Thus, temporal profiling of MSCs’ functional activity by RTCA allowed us to define the window of possible applications of CePO_4_ in vivo.

Studies on human fibroblasts, although they did not reveal a statistically significant advantage for any of the samples, showed that the greatest stimulation of fibroblasts occurred at a concentration of 10^−3^ M in all samples, with a tendency for the greatest stimulation of metabolism and proliferation to occur in the CePO_4_-II sample at a concentration of 10^−3^ M. The same sample demonstrated the best results in terms of metabolism and proliferation in our studies on human keratinocytes.

As a result of the conducted research, it can be said that to achieve the greatest stimulation of the proliferative and metabolic activity of all the cell cultures that we used in our experiment, CePO_4_-II at a concentration of 10^−3^ M should be chosen, as it stimulated the metabolism and proliferation of the cell cultures to the greatest extent.

Using the chemiluminescent method, we demonstrated the high antioxidant activity of cerium orthophosphate, which is higher than that of ascorbic acid at an equivalent concentration. Thus, we confirmed the antioxidant ability of cerium phosphate nanoparticles that was previously shown by other researchers [37,70]. Like ascorbic acid, the nanoparticles showed a dose-dependent antioxidant effect. The greatest effect was observed at 10^−2^ M and 10^−3^ M (10.2 times), while a slight effect was observed at 10^−5^ M (1.2 times).

At the same time, the authors are aware that so far, there are far more questions regarding the interaction of cerium nanocompounds with cells in living organisms than there are answers [71]. Therefore, we still have work to do to study the effects of cerium orthophosphate nanoparticles in cellular and animal models. We understand that we may encounter some difficulties that we are only beginning to understand [72]. However, this work has already begun, and its first results will be presented soon.

### Limitations and Prospects

The authors acknowledge that this work, like all scientific research, is not without limitations, which are essentially points of potential growth and development for research. Understanding a mechanism, whether it is a mechanism of disease development or one of drug action, is crucial for the targeted improvement of treatment methods. However, we are still in the early stages of this journey, hoping to find an effective treatment based on cerium phosphate nanoparticles. The in vivo studies that we have conducted are limited in scope and cannot yet be transferred directly to all types of cells, let alone multicellular organisms. Additionally, given the tendency of cerium phosphate nanoparticles to agglomerate, there may be significant issues with the stability of the nanoparticles during storage, which could potentially affect their biological properties. To solve the problems of particle agglomeration, we are conducting research on the inclusion of various nanoparticle stabilizers (excipients), such as citric acid and cellulose, and selecting their dosage to achieve the optimal benefit–effect ratio. Although the absence of cell death involved in the regeneration of skin wounds, along with high metabolic activity, may indicate the effective influence of cerium phosphate nanoparticles, including potentially on wound healing, this hypothesis can be verified in further studies, particularly in full-thickness skin wound models in animals. Our future research will focus on these issues. We have already received approval from ethical committees and conducted several promising in vivo studies. After analyzing the data, we will present our initial results.

## 4. Materials and Methods

### 4.1. Synthesis of Cerium Orthophosphate Nanoparticles

Cerium(III) orthophosphate nanoparticles were obtained by precipitation with ammonium dihydrogen phosphate from a solution of cerium(III) nitrate. In general, the synthesis of nanosized cerium(III) phosphate can be described by the following reaction equation:

Ce(NO_3_)_3_ + NH_4_H_2_PO_4_ → CePO_4_∙xH_2_O ↓ + NH_4_NO_3_ + 2HNO_3_ (1)

In all synthesis methods, cerium(III) nitrate (99.995% in terms of rare-earth elements, LANHIT LLC, Moscow, Russia) was used. The impurity composition of the initial cerium carbonate was analyzed at JSC Giredmet. As a precipitant, a solution of NH_4_H_2_PO_4_ (98%, Ruschim, Moscow, Russia) was chosen.

This work presents three modifications of the synthesis of CePO_4_, which differ in the concentrations of the initial solution of cerium nitrate and the precipitant, the pH, and the temperature of the washing water. The remaining synthesis conditions remained unchanged: the precipitation time was 7–10 min, the stirring speed was 350 rpm, and the drying temperature was 25 °C. In all cases the annealing temperature was 200 °C (as increasing the annealing temperature can significantly increase the size of the resulting particles). The other synthesis conditions for the nanosized cerium phosphate are presented in Table 4.

The precipitation #1 (CePO_4_-I) was made from the Ce(NO_3_)_3_ solution (200 mL with a CeO_2_ concentration of 125 g/L) which was placed into a reaction vessel together with 450 mL of distilled water under constant stirring (350 rpm) by addition of 350 mL of a 100 g/L NH_4_H_2_PO_4_ solution. The precipitator was drip-fed from a separating funnel into the reaction vessel for 7–10 min. The resulting volume was 1 L, resulting pH—3–4 (adjusted with an ammonia solution). The cerium (III) phosphate was filtered from the mother liquor immediately after precipitation using a Buchner funnel through a double paper filter (blue tape). Then it was washed with 1.5 L of double-distilled water at 40–50 °C. The precipitate was air-dried at 25 °C until it reached an approximate moisture content of 6% and calcined at 200 °C for three hours in a quartz cuvette.

The precipitation #2 (CePO_4_-II) was made from the Ce(NO_3_)_3_ solution (200 mL with a CeO_2_ concentration of 50 g/L) which was placed into a reaction vessel together with 200 mL of a crystallization regulator (NH_4_NO_3_) with a concentration of 375 g/L and 450 mL of distilled water under constant stirring (350 rpm) by addition of 150 mL of a 100 g/L NH_4_H_2_PO_4_ solution. The precipitator was drip-fed from a separating funnel into the reaction vessel for 7–10 min. The resulting volume was 1 L, and the resulting pH was approximately 2 (not adjusted). The cerium (III) phosphate was filtered from the mother liquor immediately after precipitation using a Buchner funnel through a double paper filter (blue tape). Then, it was washed with 3 L of double-distilled water at 20–30 °C. The precipitate was air-dried at 25 °C and calcined in a muffle furnace at 200 °C for three.

The precipitation #3 (CePO_4_-III) was made from the Ce(NO_3_)_3_ solution (200 mL with a CeO_2_ concentration of 125 g/L) which was placed into a reaction vessel together with 50 mL of distilled water under constant stirring (350 rpm) by addition of 750 mL of a 50 g/L NH_4_H_2_PO_4_ solution. The precipitator was drip-fed from a separating funnel into the reaction vessel for 7–10 min. The resulting volume was 1 L, resulting pH—approximately 2 (not adjusted). The cerium (III) phosphate was filtered from the mother liquor immediately after precipitation using a Buchner funnel through a double paper filter (blue tape). Then it was washed with 3 L of double-distilled water at 20 °C. The precipitate was air-dried at 25 °C and calcined in a muffle furnace at 200 °C for two hours.

### The Studied Samples

For the purpose of carrying out the physical and chemical examinations, samples of powders of CePO_4_, as well as aqueous suspensions, were prepared. For the purpose of carrying out the biomedical examinations on all cell lines, aqueous suspensions of CePO_4_ were prepared at concentrations of 10^−2^, 10^−3^, 10^−4^, and 10^−5^ M using sterile bidistilled water (pH 5.4–6.6 in accordance with the Russian GOST58144 standard). The concentration of CePO_4_ NPs suspension samples was determined via inductively coupled plasma mass spectrometry (ICP-MS) with an ELAN D Elan DRC-e mass spectrometer (Perkin Elmer, Shelton, CT, USA).

After calcining cerium(III) phosphate in a muffle furnace at 200 °C for 2–3 h (which guarantees the sterility of the resulting compounds), all further manipulations with samples of CePO_4_ powders for biomedical research were performed under aseptic conditions using sterile solutions. Prepared suspensions of 10 volume percent were added to the culture medium, and the actual final concentration in the wells was 10 times lower than the initial volume added.

### 4.2. Physicochemical Characterization of Nanoparticles

The following methods were used to identify the sizes of the obtained cerium phosphate nanoparticles and their morphology: transmission electron microscopy and X-ray diffraction analysis. In addition, the samples were investigated using X-ray photoelectron spectroscopy. Each sample was examined once.

Transmission electron microscopy (TEM) was performed using a JEOL JEM 2100 transmission electron microscope with a maximum accelerating voltage of 200 kV (JEOL, Akishima, Japan) in the Collective Use Center “Materials Science and Metallurgy” of the National University of Science & Technology MISIS. Particle size measurements were performed using DigitalMicrograph software (Gatan Inc, Pleasanton, CA, USA). At least 300 particles were analyzed to create particle size distribution histograms. When establishing the particle size distribution, the longitudinal and transverse dimensions of the particles were taken into account.

X-ray diffraction (XRD) of the powders was conducted using a Rigaku Ultima IV X-ray diffractometer (Rigaku, Tokyo, Japan) with Co Kα radiation (*λ* = 0.179 nm). The measurements were taken at diffraction angles of 2Θ from 10° to 80° at a scan rate of 0.1° at room temperature. The qualitative phase analysis was performed by comparing the spectra using the PDXL 2.1: Integrated software for powder diffraction software v2.

X-ray photoelectron spectroscopy (XPS) of the samples was performed on a photoelectron spectrometer OMICRON ESCA + (Scienta Omicron, Uppsala, Sweden) with an Al K*α* X-ray source (*E* = 1486.6 eV, 252 W).To compensate for the local charging of the analyzed surface, a CN-10 charge neutralizer was used with an emission current of 6 μA and a beam energy of 1 eV. The main state of C1s core level was used as a reference with a binding energy (BE) of 285 eV. The spectral information was processed using the FitXPS software v1.

### 4.3. Biomedical Research on Cell Lines

#### 4.3.1. Study of the Toxicity/Biocompatibility and Effect of CePO_4_ Nanoparticles on the Activity of Human Mesenchymal Stem Cells

##### Culture of Human Adipose-Derived Mesenchymal Stromal Cells

Two human adipose-derived MSC lines, which were previously generated in our laboratory, were used in this study. Specimens of human subcutaneous adipose tissue were collected as waste material, which was a byproduct of elective surgery from patients who signed voluntary informed consent forms. The procedures were conducted in accordance with the Declaration of Helsinki. Protocol was approved by the Ethical Committee of Pirogov Russian National Research Medical University (the Local Ethics Committee meeting No. 189 dated 10.05.2019). The enzymatic method was used for isolation and cultivation of MSCs, as previously declared [73]. Cells at passage 3 were used for experiments.

MSCs were cultured in a humidified atmosphere containing 5% CO_2_ at 37 °C on standard plastic dishes for cell culture (Corning Costar, Corning, NY, USA) in Dulbecco’s Modified Eagle Medium/Nutrient Mixture F-12 (DMEM/F12; Thermo Fisher Scientific, Waltham, MA, USA), supplemented with 100 U/mL penicillin and streptomycin, 2 mM L-glutamine (Paneco, Moscow, Russia), and 10% fetal bovine serum (One Shot FBS; Gibco, Thermo Fisher Scientific, Waltham, MA, USA) until ~70−80% confluence. The medium was changed every 3−4 d. Then MSCs were passaged with a trypsin/EDTA solution (Paneco, Moscow, Russia).

The identity of the expanded adipose-derived MSCs was confirmed using the minimal criteria proposed by the International Society for Cellular Therapy [74]. The 3rd-passage MSCs met these criteria. MSCs attached to the plastic surface were positive for CD73, CD90, and CD105 expressions and negative for CD34, CD45, and CD14 expressions. These cells were able to differentiate into adipogenic, osteogenic, and chondrogenic lineages under specific conditions, as described in [73].

##### Monitoring MSC Growth Using the xCELLigence DP System

To evaluate the MSCs’ response to application of different CePO_4_ preparations, we performed RTCA using the xCELLigence system. This technology allows for the monitoring of cells’ viability, behavior, and function by measuring the electrical impedance over time using gold microelectrode biosensors in microtiter plates, without the need for cell labeling. Adherent cells disrupt the interaction of the electrodes with the solution, increasing the impedance, which is defined as the cellular index (CI). Real-time CI changes and sigmoidal dose–response curves are displayed as graphs using RTCA software Pro. First, 50 μL of growth cell culture medium (DMEM supplemented with 10% FBS) was added to each well of E-plate 16. The E-plate 16 was then connected to the system to obtain background impedance in the absence of cells. MSCs were resuspended in the growth cell culture medium to 1.5 × 10^6^ cells/mL and serially two-fold diluted down to 350,000 cells/mL. Then, 150 μL of the resulting cell suspensions were added to the wells in order to determine the optimal cell concentration. Subsequently, the E-plate 16 was placed on the RTCA station for continuous impedance recording inside an incubator at 37 °C in a humidified atmosphere with 5% CO_2_. CI values were measured every 15 min for 12 h during cell adhesion and spreading. Then 10^−2^ M, 10^−3^ M, 10^−4^ M, and 10^−5^ M of CePO_4_ in four duplications were added to the MSCs, and CI values were measured every 30 min for 170 h. Intact MSCs were used as controls.

#### 4.3.2. Study of the Toxicity/Biocompatibility and Effect of CePO_4_ Nanoparticles on the Activity of Human Keratinocytes and Fibroblasts

In this study, we used the HaCaT and BJ hTERT cell lines. The HaCaT cell line is a spontaneously immortalized adult human keratinocyte (origin: N.N. Blokhin National Medical Research Center of Oncology, Ministry of Health of the Russian Federation, Moscow, Russia). The BJhTERT cell line is a derivative of the normal cell line, BJ, which were immortalized by transgenesis of hTERT (origin: American Type Culture Collection (ATCC, CRL-3627), Manassas, VA, USA). Cell culture was performed under standard controlled conditions in a CO_2_ incubator (Binder, Germany) at 37 °C, 5% CO_2_ in air, and controlled humidity. A detailed culture protocol is described in our previous articles [36,41].

Cerium phosphate samples in the concentration range of 10^−3^ M–10^−5^ M were added to the studied biological test systems (HaCaT and BJ hTERT) in a volume of 100 μL. The initial solvent, distilled water, was used as a control. The studied samples were added 24 h after cell passage into culture plates (SPL, Pocheon, Korea) in a complete DMEM culture medium (Paneco, Moscow, Russia), followed by coincubation for the next 72 h under standard controlled conditions. At the end of coincubation, the MTT assay and direct cell counting, including determination of the percentage of dead cells using trypan blue dye (Paneco, Moscow, Russia), were performed. The cell number and cytotoxicity assessment were performed automatically using the Countess II Automated Cell Counter (Thermo Scientific, Waltham, MA, USA) with the use of special disposable slides C100 (RWD, Shenzhen, China). As a result, the total cell concentration in a unit volume was calculated, expressing the total in the number of cells (× 10,000 cells), as well as the percentage of live and dead cells. Each sample in each group was examined in at least 7 wells. The optical density (MTT) was measured using a Multiscan spectrophotometer (Multiscan, Labsystems, Vantaa, Finland) at a wavelength of *λ* = 540 nm. The final measurement result was expressed in relative optical density (OD) units. Each sample in each group was examined in at least 10 wells. These tests for assessing the metabolic, proliferative, and cytotoxic activity of the substances were carried out according to the standard protocol described by us previously [35,40].

### 4.4. Study of Antioxidant Properties of CePO_4_ Using Chemiluminescence Method

The antioxidant activity of the best CePO_4_-II based on our research results on different cell lines was determined using a Lum-100 chemiluminometer (DISoft, Moscow, Russia) using PowerGraph 3.3.12 software. The Fenton reaction was used as the basis for the chemiluminescence reaction. In a chemiluminometer test tube, 100 μL of iron (II) sulfate solution (pure, Ruskhim, Moscow, Russia), acidified with sulfuric acid (chemically pure, Sigma Tek, Khimki, Russia) to pH = 2, at a concentration of 10^−3^ M (for iron); 100 μL of L-methionine solution (USP grade, PanEco, Moscow, Russia) at a concentration of 4 × 10^−5^ M; 2200 μL of deionized water, acidified with sulfuric acid solution to pH = 2; and 100 μL of hydrogen peroxide solution (Samaramedprom, Samara, Russia) at a concentration of 3 × 10^−3^ M were collected, and the chemiluminescence was recorded (Control group). To evaluate the antioxidant activity of CePO_4_ nanoparticles at concentrations of 10^−2^ M, 10^−3^ M, 10^−4^ M, and 10^−5^ M, the corresponding suspensions were introduced at a volume of 100 μL before adding hydrogen. The comparison antioxidant was ascorbic acid at the same concentrations.

The registration of luminescence began automatically, and no more than 1 s passed from the start of the reaction to the start of registration. The intensity of chemiluminescence was recorded in relative units (PPS—pulses per second), and the registration frequency was 1 Hz. The number of measurements for each group was at least 5 (n = 5).

For the reference experiment (control), the chemiluminescence of the Fenton reaction modified by the addition of methionine was measured for 10 min. Starting from 110 s, a plateau (termination of the reaction) was recorded.

Using the PowerGraph 3.3.12 software, the areas under the graph were calculated to characterize the total integral light sum (S) for each experiment over 120 s (from the beginning to the complete cessation of chemiluminescence). The lower the values were, the more pronounced the antioxidant properties were.

### 4.5. Statistical Analysis

The statistical program SPSS 25.0 (IBM, Armonk, NY, USA) was used to process the results of our biomedical examinations. The Shapiro–Wilk and Kolmogorov–Smirnov criteria were used to evaluate the normality of indicator distributions. For descriptive statistics of continuous quantitative indicators, which obeyed the law of normal distribution the Mean, Std. Deviation, Std. Error, 95% Confidence Interval for Mean (95CI), Minimum, Maximum were used, and for indicators, which did not follow the law of normal distribution, the median and quartiles were used. In the cell experiments, the mean value was determined in the control group. Relative to the mean value in each experiment, the percentages in the studied groups were calculated, obtaining the final figure, which was the percentage of the control. For the comparative analysis of multiple subgroups, one-factor ANOVA analysis was performed using post hoc comparisons and Dunnett’s test (for comparison with controls). Independent samples that did not follow the normal distribution were compared using the Kruskal–Wallis test, and the Bonferroni correction for multiple comparisons was taken into account when calculating the *p*-value. For all criteria and tests, differences were considered statistically significant at *p* < 0.05.

## 5. Conclusions

The main conclusions of our work are as follows:

1. Cerium(III) orthophosphate with a rhabdophane structure was obtained by precipitating ammonium dihydrogen phosphate from a cerium nitrate solution. Optimal conditions for obtaining the CePO_4_ nanopowders were established by varying the initial solution concentrations and drying and annealing temperatures. Their particle size ranges from 2 to 10 nm in the transverse direction and 20 to 50 nm in the longitudinal direction.
2. Cell line studies demonstrated a high level of safety and biocompatibility across a wide concentration range (10^−2^ or 10^−3^ to 10^−5^ M).
3. The regenerative potential of CePO_4_ nanoparticles on different cells was proven: significant enhancement of MSC proliferation at concentrations of 10^−2^–10^−3^ M within 48 h post-application and stimulation of human keratinocyte and fibroblast metabolism at concentrations of 10^−3^–10^−5^ M within 72 h post-application.
4. Under the conditions of the chemical experiment, a dose-dependent antioxidant effect of CePO_4_ nanoparticles at concentrations of 10^−2^–10^−5^ M was established, which suggests that this property will be preserved when they come into contact with living cellular objects and multicellular organisms.

Thus, a method for obtaining cerium phosphate nanoparticles with beneficial biomedical effects has been developed under industrial production conditions. The non-cytotoxicity and regenerative potential of CePO_4_ has been established in a wide range of concentrations and on different cell lines that are involved in the healing of skin wounds (mesenchymal stem cells, keratinocytes, human fibroblasts), as have their antioxidant properties.

## 6. Patents

Patent applications have been submitted (No. 2025123346, dated 28.08.2025, and No. 2025124956, dated 10.09.2025; the names of the inventions are “Method of obtaining phosphate-containing compositions based on cerium” and “Method for producing nanosized cerium phosphate”).

## Figures and Tables

**Table 1:** Results of X-ray diffraction analysis.

	CePO_4_-I	CePO_4_-II	CePO_4_-III
Group	180:P6222	152:P3121	180:P6222
Coherent scattering region, nm	6.7 ± 0.6	12.6 ± 1.2	3.51 ± 0.2
COD	9,010,969	9,015,938	9,010,969

**Table 2:** Results of the X-ray photoelectron spectroscopy analysis of the samples.

Binding Energy, eV	CePO_4_-I	CePO_4_-II	CePO_4_-III	CePO_4_ [Database Reference Number]
Ce(3d_3/2_)	904.47	904.86	904.01	904.00
Ce(3d_5/2_)	885.87	885.98	885.81	885.40
P(2p_3/2_)	133.47	133.67	133.41	133.30
O(1s)	531.37	531.37	531.41	531.00

Database: NIST X-ray Photoelectron Spectroscopy Database (SRD 20), Version 5.0.

**Table 3:** Values of integral light sums (S) for the studied samples (S, kPPS⋅s).

Control		Ascorbic Acid	CePO_4_
133.82 ± 6.05	Drug, 10^−2^ M	12.18 ± 0.61	2.99 ± 0.13
	Drug, 10^−3^ M	58.48 ± 2.54	13.11 ± 0.45
	Drug, 10^−4^ M	67.14 ± 3.27	54.06 ± 2.28
	Drug, 10^−5^ M	120.45 ± 4.95	113.70 ± 4.84

**Table 4:** The conditions and results of the CePO_4_ NP synthesis (concentrations are given in a total volume of 1 L).

Conditions	CePO_4_-I	CePO_4_-II	CePO_4_-III
C_Ce(NO3)3_, g/L (by CeO_2_ content)	25	10	25
C_(NH4H2PO4)_, g/L	35	15	37.5
C_(NH4NO3)_, g/L	–	75	–
pH	3–4	2–3	2
T_H2O_, °C	40–50	30–20	15–20
Exposure time; h	3	3	2
Yield of finished product, % of theoretical	97.5	95	98.5

## Data Availability

Data can be found within the article, and primary data are also available from corresponding authors.
